# Distinctive and Complementary Roles of Default Mode Network Subsystems in Semantic Cognition

**DOI:** 10.1523/JNEUROSCI.1907-23.2024

**Published:** 2024-04-08

**Authors:** Ximing Shao, Katya Krieger-Redwood, Meichao Zhang, Paul Hoffman, Lucilla Lanzoni, Robert Leech, Jonathan Smallwood, Elizabeth Jefferies

**Affiliations:** ^1^Department of Psychology, University of York, York, YO10 5DD, United Kingdom; ^2^CAS Key Laboratory of Behavioural Science, Institute of Psychology, Chinese Academy of Sciences, Beijing 100101, China; ^3^Department of Psychology, University of Chinese Academy of Sciences, Beijing 100049, China; ^4^School of Philosophy, Psychology and Language Sciences, University of Edinburgh, Edinburgh EH8 9JZ, United Kingdom; ^5^Centre for Neuroimaging Science, Institute of Psychiatry, Psychology and Neuroscience, King’s College London, London SE5 9RT, United Kingdom; ^6^Department of Psychology, Queen’s University, Kingston, Ontario K7L 3N6, Canada

**Keywords:** activation and deactivation, default mode network, fMRI, functional connectivity, semantics

## Abstract

The default mode network (DMN) typically deactivates to external tasks, yet supports semantic cognition. It comprises medial temporal (MT), core, and frontotemporal (FT) subsystems, but its functional organization is unclear: the requirement for perceptual coupling versus decoupling, input modality (visual/verbal), type of information (social/spatial), and control demands all potentially affect its recruitment. We examined the effect of these factors on activation and deactivation of DMN subsystems during semantic cognition, across four task-based human functional magnetic resonance imaging (fMRI) datasets, and localized these responses in whole-brain state space defined by gradients of intrinsic connectivity. FT showed activation consistent with a central role across domains, tasks, and modalities, although it was most responsive to abstract, verbal tasks; this subsystem uniquely showed more “tuned” states characterized by increases in both activation and deactivation when semantic retrieval demands were higher. MT also activated to both perceptually coupled (scenes) and decoupled (autobiographical memory) tasks and showed stronger responses to picture associations, consistent with a role in scene construction. Core DMN consistently showed deactivation, especially to externally oriented tasks. These diverse contributions of DMN subsystems to semantic cognition were related to their location on intrinsic connectivity gradients: activation was closer to the sensory-motor cortex than deactivation, particularly for FT and MT, while activation for core DMN was distant from both visual cortex and cognitive control. These results reveal distinctive yet complementary DMN responses: MT and FT support different memory-based representations that are accessed externally and internally, while deactivation in core DMN is associated with demanding, external semantic tasks.

## Significance Statement

We delineate the functional organization of default mode network (DMN) in semantic cognition, examining effects of perceptual coupling versus decoupling, input modality (visual/verbal), domain (social/spatial), and control demands across DMN subsystems in four fMRI datasets. These subsystems played complementary roles in semantic cognition related to their locations on gradients of intrinsic connectivity. Medial temporal and frontotemporal subsystems supported visuospatial and abstract conceptual information, respectively, across both internally and externally focused tasks, while deactivation in core DMN was associated with focused and externally oriented semantic states. We conclude that both content and process are relevant to the functional architecture of DMN in semantic cognition.

## Introduction

Default mode network (DMN)—with distributed components across medial and lateral frontal, parietal, and temporal cortex—deactivates in attention-demanding tasks (Raichle et al., 2001; Greicius et al., 2003) and is anticorrelated with dorsal attention network (Greicius et al., 2003; Fox et al., 2005). However, DMN can couple with visual inputs (Zhang et al., 2019, 2022), and it supports both perceptually decoupled states (Christoff et al., 2009; Konu et al., 2020) and aspects of externally oriented cognition, including semantic processing (Binder et al., 2009; Krieger-Redwood et al., 2016) and working memory (Spreng et al., 2014; Vatansever et al., 2015; Murphy et al., 2018). DMN deactivation might support semantic cognition: connectivity with control regions increases during demanding conceptual decisions (Krieger-Redwood et al., 2016) and multivariate analysis reveals semantic goal representations (Wang et al., 2021) even in “task-negative” regions.

A contemporary topographical perspective suggests the functions of DMN might reflect its distance from unimodal cortex (Margulies et al., 2016; Smallwood et al., 2021). This view is supported by whole-brain decompositions of intrinsic connectivity, termed “gradients,” which capture key features of cortical organization (Margulies et al., 2016; Hong et al., 2020; McKeown et al., 2020). The principal gradient, explaining the most variance, captures the separation of heteromodal DMN from the sensory-motor cortex (Margulies et al., 2016) and correlates with physical distance from sensory-motor landmarks on the cortical surface. The second gradient relates to the distinction between the visual and motor cortex, while the third gradient reflects the division within heteromodal cortex between controlled and less controlled responses. DMN's position on the principal gradient far from the sensory-motor cortex might support perceptually decoupled states. However, DMN's position at the end of multiple processing streams might also facilitate the coordination and abstraction of higher-order representations.

DMN also contains subsystems associated with different cognitive processes (Andrews-Hanna, 2012; Andrews-Hanna et al., 2014): (1) medial temporal (MT) DMN is linked to episodic memory and scene construction; (2) core DMN with self-referential and perceptually decoupled cognition; while (3) frontotemporal (FT) DMN is thought to support abstract, semantic, and social cognition (Andrews-Hanna, 2012; Andrews-Hanna and Grilli, 2021). These subnetworks suggest a complex functional organization, the principles of which are still not fully understood. While tasks eliciting FT activation are often semantic and tasks eliciting MT and core activation often involve episodic retrieval, there are multiple confounds in this contrast: semantic cognition is typically more perceptually coupled (involving access to meaning from visual inputs), abstract (involving verbal or categorical representations, as opposed to reconstructions of places and events), and controlled (involving more ambiguous decisions about information in the absence of recent exposure; cf. Vatansever et al., 2021). These dimensions might affect activation and deactivation in DMN subsystems in distinct ways, with all three subnetworks supporting semantic cognition if task demands are configured appropriately (Humphreys et al., 2015; Krieger-Redwood et al., 2016; Vatansever et al., 2021). Moreover, the core DMN network remains controversial since MT and FT subsystems show interdigitated connectivity in core regions (Braga and Buckner, 2017): this network might reflect inadequate spatial resolution and/or the integration of informational states across the other subsystems.

This study delineates the role of DMN subsystems in semantic cognition, examining effects of perceptual coupling versus decoupling (in a comparison of reading and autobiographical memory retrieval), modality (words/pictures), abstractness, feature type (valance/spatial), and control demands. We examine both activating and deactivating voxels in each subsystem for each participant, since deactivation might support cognition by suppressing task-irrelevant information (Amedi et al., 2005; Azulay et al., 2009). We localize the activating and deactivating voxels for each subnetwork in gradient space, as the first three connectivity gradients relate to frequently confounded cognitive dimensions in studies of DMN function, including abstraction (principal gradient), the balance of sensory-motor inputs (second gradient), and control demands (gradient three). We hypothesize that functional distinctions between DMN subsystems will relate to the connectivity of their activating and deactivating voxels to heteromodal, modality-specific, and control networks, captured by these gradients.

## Materials and Methods

The present study investigated DMN activity in five independent published fMRI datasets. The key materials and methods are described below but additional details about each dataset are available in previous publications (Study 1: Zhang et al., 2022; Study 2: Krieger-Redwood et al., 2015; Study 3: Hoffman et al., 2015; Study 4: Lanzoni et al., 2020; Study 5: Shao et al., 2022).

### Participants

The samples included: 29 participants (Study 1: mean age ± SD = 20.14 ± 1.26 years, 6 males), 22 participants (Study 2: 23 participants recruited, one removed due to low accuracy, mean age = 23.2 ± 2.9 years, 16 males), 19 participants (Study 3: 20 participants recruited, one removed due to image artifacts; mean age = 25 years, 11 males), 26 participants (Study 4: 27 participants recruited, one excluded due to no behavioral responses being recorded; mean age = 21.5 ± 2.9 years, 9 males), and 176 participants (Study 5: 207 participants recruited, 31 excluded: 25 with missing behavioral data, 2 with missing or incorrectly recorded imaging data, and 4 during preprocessing because they exceeded 0.3 mm motion, 20% invalid scans, and/or *z* > 2 mean global signal change; mean age = 20.57, 62 males). All participants were right-handed native English speakers and had normal or corrected-to-normal vision. None had any history of neurological impairment, diagnosis of learning difficulty, or psychiatric illness. All participants provided written informed consent prior to taking part. All studies were approved by the local ethics committee.

### Procedure

#### Study 1: reading and autobiographical memory

##### Study 1 tasks

**Figure 1.**
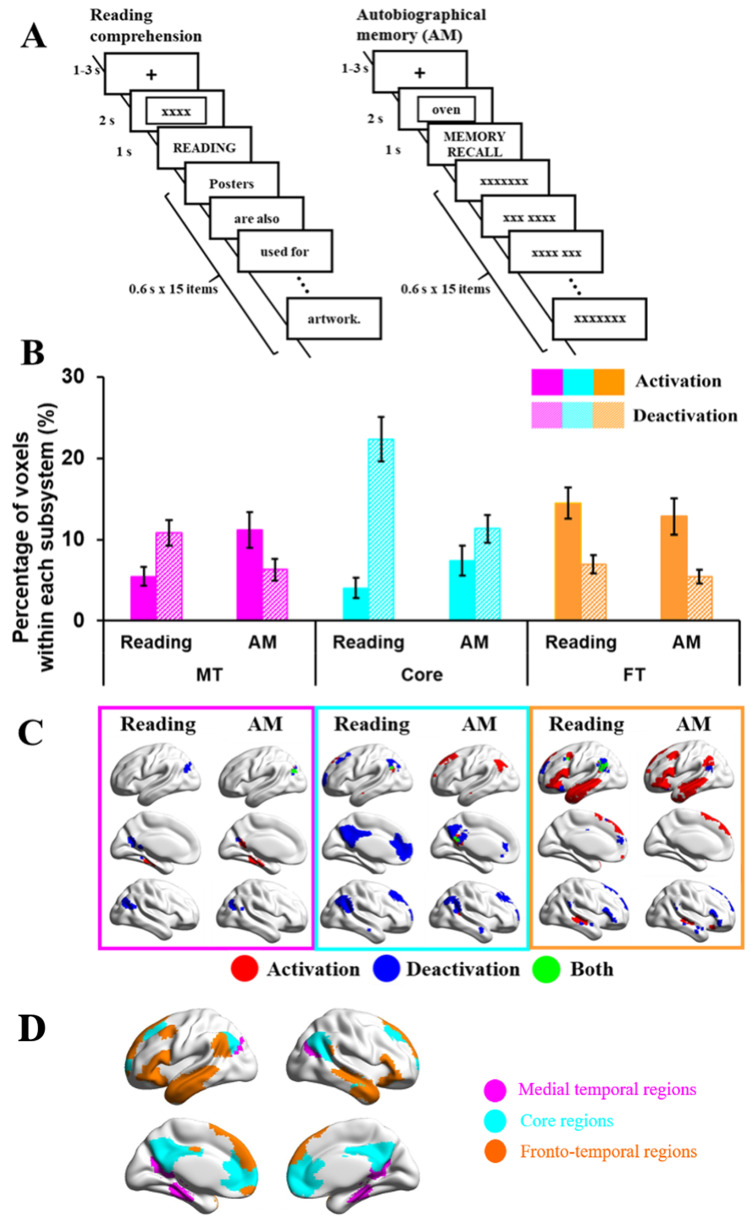
***A***, Example of task procedures of reading or autobiographical memory (recall) tasks (Study 1). ***B***, Percentage of activating or deactivating voxels in reading or autobiographical memory (recall) tasks. The three colors represent the percentage of voxels extracted from three subsystems (pink, medial temporal regions; cyan, core regions; orange, frontotemporal regions). Solid bars represent the percentages of activating voxels, and grid bars represent the percentage of deactivating voxels. Error bars represent one standard error. ***C***, Regions of activating voxels (in red color), deactivating voxels (in blue color), and the overlapping regions of both activating and deactivating voxels (i.e., regions showed activation in several participants and deactivation in another group of participants, in green color). ***D***, Maps of three subsystems (pink, medial temporal regions; cyan, core regions; orange, frontotemporal regions).

The first study compared DMN recruitment during tasks that involved conceptual access driven by visual inputs (in reading comprehension) and internally directed memory retrieval (in autobiographical memory recall). Testing occurred across 2 consecutive days (see Fig. 1*A* for task design). On Day 1, participants generated their own personal memories from cue words (i.e., Party) outside the scanner. They were asked to identify specific events that they were personally involved in and to provide as much detail about these events as they could, including when and where the event took place, who was involved, what happened, and the duration. They typed these details into a spreadsheet to ensure comparable information was recorded for the different cue words.

On the following day, participants were asked to read sentences for comprehension or to recall their generated personal memories inside the scanner. In reading trials, sentences were presented word by word, after either (1) an autobiographical memory cue word (e.g., Party), creating conflict between reading and personal memory retrieval, or (2) a letter string (e.g., XXX) allowing reading to take place in the absence of conflict from autobiographical memory. We controlled the duration of the sentences by presenting the words on 15 successive slides, combining short words or articles and conjunctions together with nouns on a single slide. In memory recall trials, participants were asked to recall autobiographical memories during the presentation of either (1) meaningful yet unrelated sentences, creating conflict from task-irrelevant patterns of semantic retrieval, or (2) letter strings (XXX) allowing autobiographical memory to take place without distracting semantic input. A baseline condition involved presenting meaningless letter strings (i.e., xxxxx) in the absence of a task.

As shown in Figure 1*A*, each trial started with a fixation cross (1–3 s) in the center of the screen. Then, either an autobiographical memory cue word or a letter string appeared for 2 s. During the presentation of the cue word, participants were asked to recall their personal memories related to this item. Next, the task instruction (i.e., READING or MEMORY RECALL) was presented for 1 s. Following that, words from sentences or letter strings were presented, with each one lasting 600 ms. On memory recall trials, participants were asked to keep thinking about their autobiographical memory, in as much detail as possible, until the end of the trial.

##### Study 1 scan parameters

Structural and functional data were acquired using a 3 T GE HDx Excite MRI scanner utilizing an eight-channel phased array head coil. Structural MRI acquisition in all participants was based on a T1-weighted 3D fast spoiled gradient-echo sequence [repetition time (TR),  7.8 s; echo time (TE), minimum full; flip angle, 20°; matrix size, 256 × 256; 176 slices; voxel size, 1.13 × 1.13 × 1 mm^3^]. The task-based activity was recorded using single-shot 2D gradient-echo-planar imaging sequence with TR, 3 s; TE, minimum full; flip angle, 90°; matrix size, 64 × 64; 60 slices; and voxel size, 3 × 3 × 3 mm^3^.

##### Study 1 preprocessing and individual-level analysis of fMRI data

All functional and structural data were preprocessed using a standard pipeline and analyzed via the FMRIB Software Library (FSL version 6.0, www.fmrib.ox.ac.uk/fsl). Individual T1-weighted structural brain images were extracted using FSL's Brain Extraction Tool (BET). Structural images were linearly registered to the MNI152 template using FMRIB's Linear Image Registration Tool (FLIRT). The first three volumes of each functional scan were removed in order to minimize the effects of magnetic saturation. The functional neuroimaging data were analyzed using FSL's FMRI Expert Analysis Tool (FEAT). We applied motion correction using MCFLIRT (37), slice timing correction using Fourier space time-series phase-shifting (interleaved), spatial smoothing using a Gaussian kernel of FWHM 6 mm, and high-pass temporal filtering (sigma = 100 s) to remove temporal signal drift. In addition, motion scrubbing (using the fsl_motion_outliers tool) was applied to exclude volumes that exceeded a framewise displacement threshold of 0.9.

The preprocessed time-series data were modeled using a general linear model, using FMRIB's Improved Linear Model (FILM) correcting for local autocorrelation. Nine explanatory variables (EVs) of interest and nine of no interest were modeled using a double-Gaussian hemodynamic response gamma function. The nine EVs of interest were as follows: Reading (1) without and (2) with conflict from memory recall, Autobiographical memory retrieval (3) with and (4) without conflict from semantic input, (5) Letter String Baseline, (6–9) Task Focus effect for each of the four experimental conditions as a parametric regressor. Our EVs of no interest were as follows: (10) Memory cue words and (11) Letter strings before the presentation of task instructions, Task instructions for Reading (12) without and (13) with conflict (this separation of the reading task instruction was based on the consideration that some recall or task preparation was likely to be occurring due to the presentation of autobiographical memory cues), plus task instructions for (14) Memory Recall and (15) Letter String baseline conditions. Other EVs of no interest were as follows: (16) Fixation (the inter-stimulus fixations between the sentences or letter strings and the ratings questions), (17) Responses to catch trials (which included all time points with responses across conditions), and (18) Rating decision periods (including all the ratings across experimental conditions). EVs for each condition commenced at the onset of the first word of the sentence or the first letter string, with EV duration set as the presentation time (9 s). The parametric EVs for the effect of Task Focus during the target had the same onset time and duration as the EVs corresponding to the four experimental trials, but in addition included the demeaned Task Focus ratings value as a weight. The fixation period between the trials provided the implicit baseline. We examined the main effects of Task and Conflict for both the main experimental conditions compared with the implicit baseline, which allowed us to identify the activation and deactivation in each task.

#### Study 2: word and picture semantic judgments

##### Study 2 tasks

**Figure 2.**
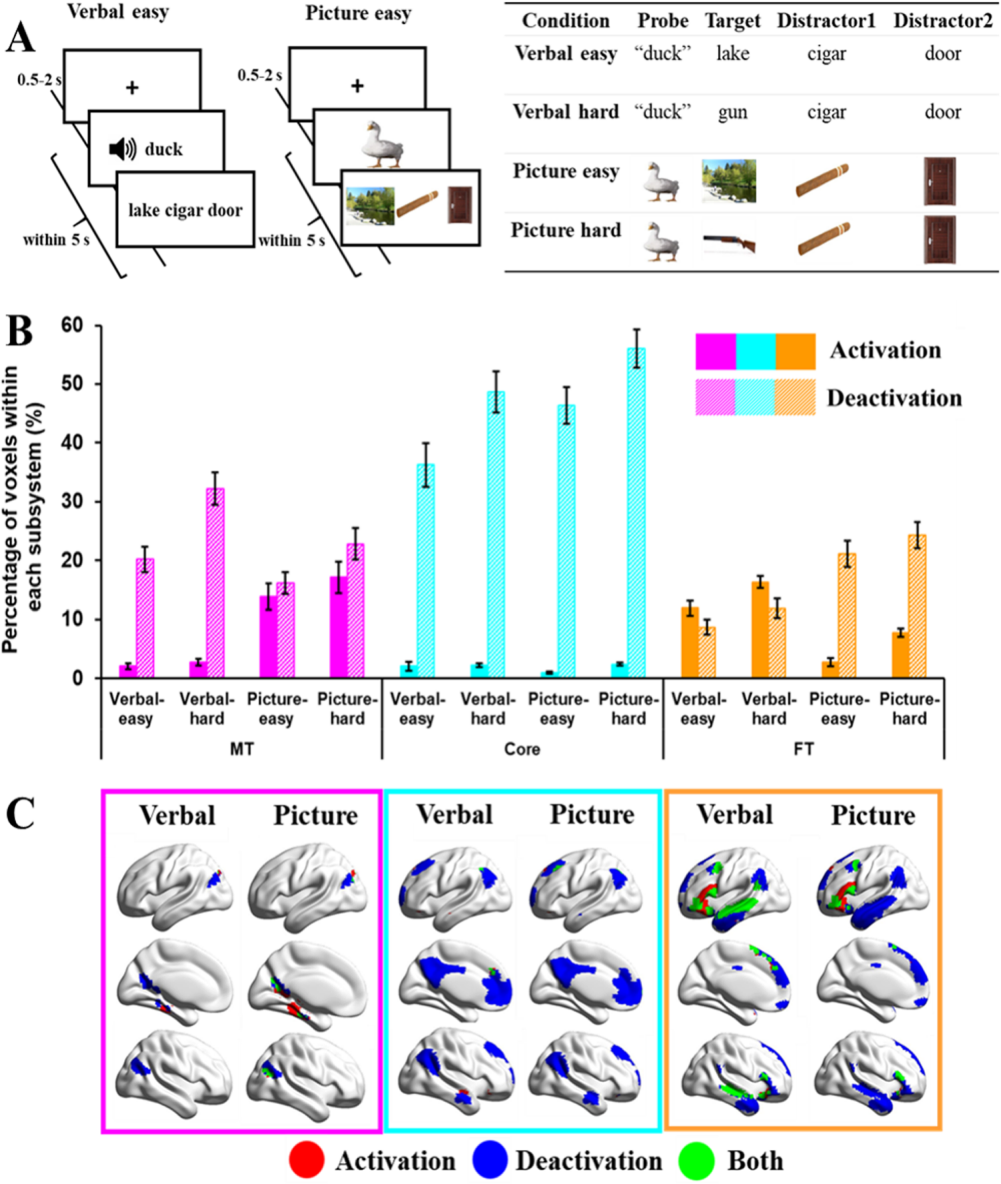
***A***, Example of task procedures of word or picture matching tasks (Study 2). ***B***, Percentage of activating or deactivating voxels in verbal or picture matching tasks. The three colors represent the percentage of voxels extracted from three subsystems (pink, medial temporal regions; cyan, core regions; orange, frontotemporal regions). Solid bars represent the percentages of activating voxels, and grid bars represent the percentage of deactivating voxels. Error bars represent one standard error. ***C***, Regions of activating voxels (in red color), deactivating voxels (in blue color), and the overlapping regions of both activating and deactivating voxels (i.e., regions showed activation in several participants, and deactivation in another group of participants, in green color).

The second study compared DMN recruitment across semantic tasks involving words and pictures and manipulated the difficulty of these decisions by contrasting strong and weak associations. A three alternative forced choice (3AFC) format was used for all conditions (see Fig. 2*A* for example stimuli and task design). The verbal task involved auditory presentation of a probe word, and response options presented as written words. The picture task used photographs of the probes, targets, and distracters.

Participants made easy and hard associative judgments: they were presented with a spoken word or picture probe, together with three word/picture response options on the screen and instructed to select the item most strongly related to the probe. The probes and targets either shared a strong association (for easy trials) or a weak association (for more difficult trials). For example, an easy association might involve the probe “duck” and three answer choices such as lake–cigar–door. A harder trial would require participants to link “duck” with gun—an association that is less frequently encountered. Strong associations are thought to be retrieved relatively automatically, since the probe establishes a context that strongly anticipates the target; in contrast, for weak associations, control processes are needed to focus retrieval on nondominant semantic features that are relevant to the linking context (e.g., a duck can be hunted; a gun is used for hunting; Jefferies, 2013; Davey et al., 2016; Lambon Ralph et al., 2017).

As shown in Figure 2*A*, each trial started with a fixation screen for a jittered interval (500–2,000 ms) followed by the trial (probe and options). Participants were required to make a response, which triggered the next trial; if no response was given after 5 s, the experiment moved onto the next trial.

##### Study 2 scan parameters

Structural and functional data were acquired with a GE 3T HDx Excite MRI scanner at the York Neuroimaging Centre (YNiC), in a single scanning session. A Magnex, 8-channel, gradient insert head coil with a birdcage and radio frequency coil tuned to 127.4 MHz was used. A gradient-echo EPI sequence was used to collect data from 39 contiguous axial slices (TR, 3 s; TE, 25 ms; FOV, 260 mm^2^; matrix size, 128 × 128; slice thickness, 3.5 mm). The functional data were coregistered onto structural T1-weighted images with a resolution of 1 × 1 × 1 mm (TR, 8.03; TE, 3.07 ms; FOV, 290 × 290 176 mm; matrix size, 256 × 256 × 176; slice thickness, 1.13 × 1.13 × 1 mm). Functional data were additionally coregistered to T1-weighted FLAIR images (5.6 × 5.6 × 3.5 mm), taken in the same plane as the EPI slices with interleaved slice acquisition.

##### Study 2 preprocessing and individual-level analysis of fMRI data

We used an event-related design for all of the analyses (i.e., to examine the effects of both difficulty and task), even though the various tasks were presented in mini-blocks. Only accurate responses were used in the analysis. All first-level and higher-level analyses were run using FEAT version 5.98, in FSL (www.fmrib.ox.ac.uk/fsl). Prior to inferential statistical analysis, the following preprocessing was applied: individual brain extraction (BET) to remove nonbrain material from images for coregistration of the functional data, MCFLIRT motion correction (using fMRIB's Linear Registration Tool; Jenkinson et al., 2002), slice timing correction using Fourier space time-series phase shifting (Sinc interpolation with a Hanning-windowing kernel), FWHM 6.0 mm spatial smoothing (Gaussian Kernel), and high-pass temporal filtering (Gaussian-weighted least-squares straight line fitting, with sigma = 100 s). We used FILM nonparametric estimation of time-series autocorrelation (FILM; FMRIB's Improved Linear Model) to fit the model to the data, on all lower-level analyses. FSL's canonical gamma HRF along with a temporal derivative was used to model the HRF response. The first two volumes were removed to allow for T1 saturation effects. To analyze the data at the group level, we entered lower level FEAT directories into a higher level FMRIB'S Local Analysis of Mixed Effects (FLAME) Bayesian mixed effects analysis (Beckmann et al., 2003; Woolrich et al., 2004; Woolrich, 2008). *Z* (Gaussianized T/F) statistic images were thresholded using clusters determined by *Z* > 2.3 and a (corrected) cluster significance threshold of *p* < 0.05 (Worsley, 2001). Names of brain areas reported are labeled according to the Harvard-Oxford Cortical Structural Atlas, Talairach Daemon, and the Juelich Histological Atlas built into the FSLView software library.

Each task and condition was modeled separately using event-based EVs which were convolved to the hemodynamic response function (gamma function). We used a variable-epoch model as recommended by Grinband et al. (2008) to capture effects of time-on-task within each EV: the hemodynamic response function was aligned to the beginning of each correct trial and lasted for the duration of the event. Incorrect/removed trials were modeled as a separate EV; therefore, any data not modeled was included as rest. Several contrasts were run (11 in total): a contrast against rest/baseline was conducted for each of the six conditions (nonsemantic easy, nonsemantic hard, semantic verbal easy, semantic verbal hard, semantic picture easy, semantic picture hard), the hard version of each judgment type was contrasted against the corresponding easy version (nonsemantic hard–nonsemantic easy, semantic verbal hard–semantic verbal easy, etc.), and two contrasts examining modality/task were included (semantic verbal–rhyme; semantic picture–semantic verbal).

#### Study 3: abstract/concrete word synonym judgments

##### Study 3 tasks

**Figure 3.**
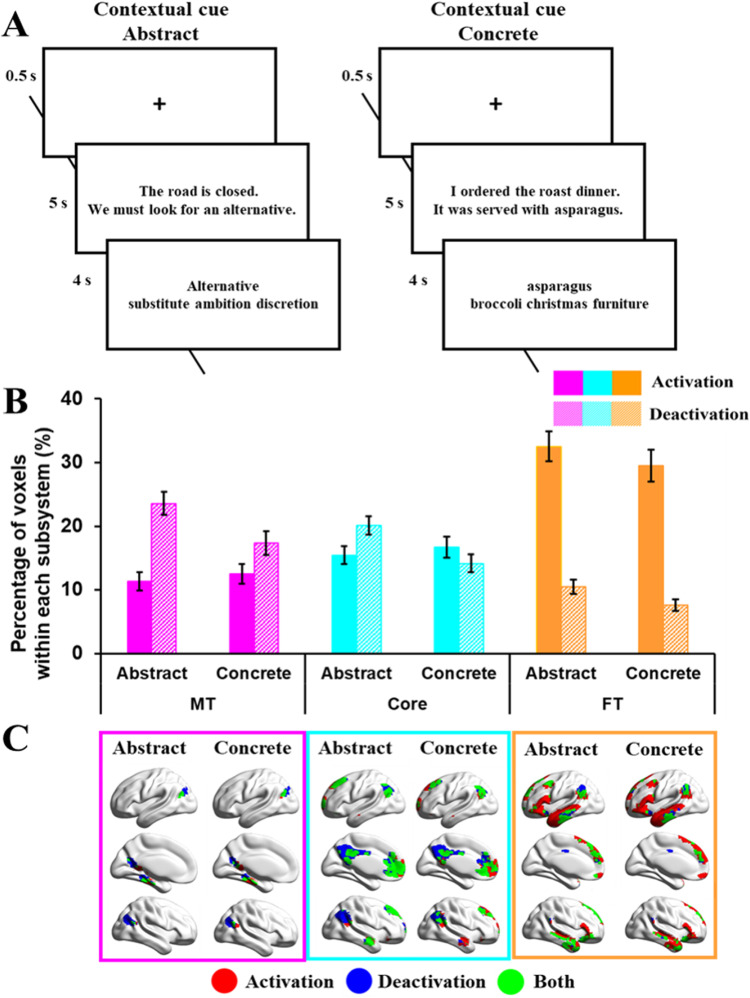
***A***, Example of task procedures of abstract or concrete tasks (Study 3). ***B***, Percentage of activating or deactivating voxels in abstract or concrete tasks. The three colors represent the percentage of voxels extracted from three subsystems (pink, medial temporal regions; cyan, core regions; orange, frontotemporal regions). Solid bars represent the percentages of activating voxels, and grid bars represent the percentage of deactivating voxels. Error bars represent one standard error. ***C***, Regions of activating voxels (in red color), deactivating voxels (in blue color), and the overlapping regions of both activating and deactivating voxels (i.e., regions showed activation in several participants, and deactivation in another group of participants, in green color).

The third study examined DMN responses to abstract and concrete concepts in a synonym judgment task (see Fig. 3*A* for task design and OSF for example stimuli, https://osf.io/vtuh4/). This study consisted of 200 trials (100 concrete and 100 abstract words); imageability was significantly higher for concrete than abstract words (*t* = 82; *p* < 0.001). On each trial, participants were presented with a written probe word with three choices below it (a semantically related target and two unrelated foils), and the probe word, target word, and two distractors had similar imageability ratings (i.e., abstractness) within each trial. They were asked to select the word that was most similar in meaning to the probe. Prior to each decision, participants were presented with a written cue consisting of two short sentences. On half of the trials, the cue ended with the probe word and placed it in a meaningful context (contextual cue condition). On the remaining trials, the cue did not contain the probe and was not related in meaning to the subsequent judgment (irrelevant cue condition). Participants were unaware when reading the cue whether it would be helpful for their next decision.

As shown in Figure 3*A*, each trial began with a fixation cross presented in the center of the screen for 500 ms, followed by the cue. Participants were instructed to read the cue carefully and to press a button on the response box when they had finished reading. The cue remained on screen for 5 s. The judgment probe and three choices were then presented and participants responded by pressing one of three buttons on a response box held in their right hand. The stimuli remained on screen for 4 s, at which point the next trial began. Stimuli were presented in blocks of two trials (total duration, 19 s) with the two trials in each block being taken from the same experimental condition. There were 150 blocks in total, and blocks from different conditions were presented in a pseudorandom order. A fixation block of 19 s, in which no stimuli were presented, occurred after every five blocks of task.

##### Study 3 scan parameters

Images were acquired on a 3 T Philips Achieva scanner using an eight element SENSE head coil with a sense factor of 2.5. A spin-echo imaging sequence, combined with a postacquisition distortion–correction, was employed to improve signal quality in the vATL (Embleton et al., 2010). The spin-echo EPI sequence included 31 slices covering the whole brain with TE, 70 ms; TR, 3200 ms; flip angle, 90°; 96 × 96 matrix; reconstructed in-plane resolution, 2.5 × 2.5 mm; and slice thickness 4.0 mm. Following the standard method for distortion-corrected spin-echo fMRI (Embleton et al., 2010), the images were acquired with a single direction *k* space traversal and a left–right phase encoding direction. In between the two functional runs, a brief “prescan” was acquired, consisting of 10 volumes of dual direction *k* space traversal SE EPI scans. These scans were used in the distortion correction procedure. In addition, a high-resolution T1-weighted 3D turbo field echo inversion recovery image was acquired (TR, 8,400 ms; TE, 3.9 ms; flip angle, 8°; 256 × 205 matrix reconstructed to 256 × 256; reconstructed resolution, 0.938 × 0.938 mm; and slice thickness of 0.9 mm; SENSE factor, 2.5) with 160 slices covering the whole brain. This image was used for spatial normalization.

##### Study 3 preprocessing and individual-level analysis of fMRI data

Analysis was carried out using SPM8. The motion and distortion-corrected images for each participant were first coregistered to their T1 structural scan. Spatial normalization of the T1 scans into Montreal Neurological Institute (MNI) space was computed using DARTEL (Ashburner, 2007), and the resulting transformation applied to the functional images, which were resampled to 2 × 2 × 2 mm voxel size and smoothed with an 8 mm FWHM Gaussian kernel. At this point, temporal signal-to-noise (TSNR) maps were generated for each participant by dividing the mean signal in each voxel by its standard deviation (Murphy et al., 2007). TSNR exceeded 80 in ventral temporal regions. Unlike gradient-echo fMRI, which shows a pronounced drop in TSNR in ventral temporal regions relative to the rest of the brain, TSNR in the ventral temporal lobes was comparable with that in frontal and superior temporal regions. The data were treated with a high-pass filter with a cutoff of 190 s and analyzed using a general linear model. At the first level, each of the five stimulus conditions was modeled with a separate regressor (concrete-context, concrete-irrelevant, abstract-context, abstract-irrelevant, and number baseline). Blocks were modeled with a boxcar function convolved with the canonical hemodynamic response function. Motion parameters were entered into the model as covariates of no interest. Parameter estimates were subjected to several analyses, each targeted at a specific hypothesis.

#### Study 4: emotional/spatial cues

##### Study 4 tasks

**Figure 4.**
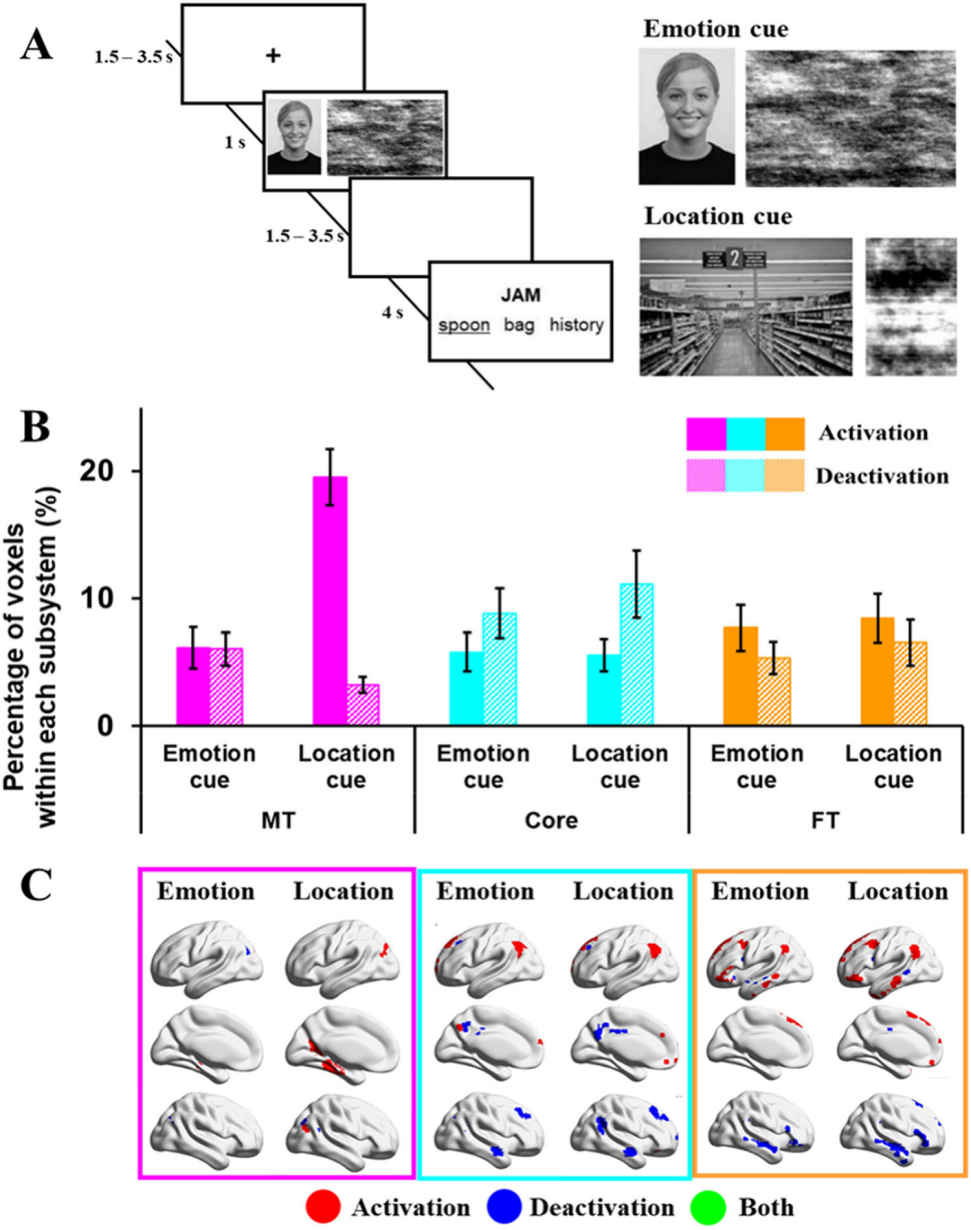
***A***, Example of task procedures of emotional or spatial cueing tasks (Study 4). ***B***, Percentage of activating or deactivating voxels in emotional or spatial cueing tasks. The three colors represent the percentage of voxels extracted from three subsystems (pink, medial temporal regions; cyan, core regions; orange, frontotemporal regions). Solid bars represent the percentages of activating voxels, and grid bars represent the percentage of deactivating voxels. Error bars represent one standard error. ***C***, Regions of activating voxels (in red color), deactivating voxels (in blue color), and the overlapping regions of both activating and deactivating voxels (i.e., regions showed activation in several participants, and deactivation in another group of participants, in green color).

The fourth study examined semantic judgments about ambiguous words preceded by portrayals of facial emotions and spatial locations (see Fig. 4*A* for task design and example stimuli). English homonyms with more than one meaning were selected as stimuli; they had different interpretations associated with different facial expressions (e.g., jam with traffic is associated with frustration while jam with strawberry is associated with pleasure) but also different locations (e.g., motorway for traffic jam and supermarket for strawberry jam). Four target words were generated for each probe, two for each interpretation. For instance, the probe jam appeared in four trials, twice paired with a target referring to traffic (jam-horn or jam-delay) and twice paired with a target referring to the alternative interpretation (jam-spoon or jam-bread).

Pictures of facial emotional expressions and spatial locations were used to prime the relevant meaning of the homonym. Each picture was used only once across the experiment, so that participants could not predict the subsequent probe word from the cue. Images of facial expressions were chosen from the Radboud Faces Database (Langner et al., 2010) and included eight basic emotions: happy, angry, sad, disgusted, contemptuous, surprised, neutral, and fearful. Pictures of spatial contexts were downloaded from Google images.

The experiment included two-cue (emotion and location), one-cue (either emotion or location), and no-cue (scrambled images) trials. The emotion and location cues were presented simultaneously in the two-cue condition, while for the one-cue conditions, images were paired with a meaningless scrambled image. The position of the emotion and location cues on the screen (on the left or right-hand side) was counterbalanced within each run. In the no-cue condition, two scrambled images were presented. We used this study to clarify the effect of emotion in driving DMN function; as such, only the one-cue condition was relevant because each trial contained either an emotion or location cue, whereas the two-cue condition contained both an emotion and a location cue for each trial (therefore, the two-cue condition does not separate the two dimensions).

As shown in Figure 4*A*, each trial began with a fixation cross (1,500–3,000 ms) followed by cue images for 1,000 ms, and then a blank screen (1,500–3,000 ms). Following this, a probe word was presented above a target word and two unrelated distracters, triggering the onset of the decision-making period. The probe and choices remained visible for a fixed interval of 4,000 ms. The assignment of the emotion-related and location-related distractors to the different conditions was counterbalanced within participants, such that each probe appeared twice with an emotion-related distractor and twice with a location-related distractor.

##### Study 4 scan parameters

Structural and functional data were acquired with a GE 3 T HDx Excite MRI scanner. Structural MRI data acquisition in all participants was based on a T1-weighted 3D fast spoiled gradient-echo sequence (TR, 7.8 ms; TE, minimum full; flip angle, 20°; matrix size, 256 × 256; 176 slices; voxel size, 1.13 × 1.13 × 1 mm). A gradient-echo EPI sequence was used to collect functional data from 60 interleaved bottom-up axial slices aligned with the temporal lobe (TR, 3 s; TE, 18.9 ms; FOV, 192 × 192 × 180 mm; matrix size, 64 × 64; slice thickness, 3 mm; slice gap, 3 mm; voxel size, 3 × 3 × 3 mm^3^; flip angle, 90°). An intermediary FLAIR scan with the same orientation as the functional scans was collected to improve the coregistration between subject-specific structural and functional scans.

##### Study 4 preprocessing and individual-level analysis of fMRI data

FMRI data processing was carried out using FEAT version 6.0, part of FSL (FMRIB's Software Library, www.fmrib.ox.ac.uk/fsl). Registration of the high resolution structural to standard space (MNI) was carried out using FLIRT (Jenkinson and Smith, 2001; Jenkinson et al., 2002). Preprocessing of the functional image included motion correction using MCFLIRT (Jenkinson et al., 2002), slice timing correction using Fourier space time-series phase-shifting (interleaved), nonbrain removal using BET (Smith, 2002), spatial smoothing using a Gaussian kernel of FWHM 5 mm, grand-mean intensity normalization of the entire 4D dataset by a single multiplicative factor, and high-pass temporal filtering (Gaussian-weighted least-squares straight line fitting, with sigma = 50.0 s). A semantic decision model with an event-related design was built to look for brain changes during semantic decisions following different levels of cueing. The semantic decision model included 8 EVs: correct semantic decisions following each of the 4 experimental conditions (0 cues, 1 cue emotion, 1 cue location, 2 cues), nonsemantic trials where strings of “Xs” were presented, remaining time in the semantic trials after making a decision before the start of a new trial, cue presentation period (combining all the cue presentation events, irrespective of the cue condition), and incorrect semantic trials.

#### Study 5: resting-state scan

Participants took part in a 9 min resting-state fMRI scan. They were instructed to focus on a fixation cross with their eyes open, and not to think about anything in particular. A structural scan was also obtained in the same session.

##### Study 5 scan parameters

Structural and functional MRI data were acquired on a 3T GE HDx Excite MRI scanner, equipped with an eight-channel phased array head coil at the York Neuroimaging Centre, University of York. For each participant, structural MRI was acquired based on a sagittal isotropic 3D fast spoiled gradient-recalled echo T1-weighted structural scan (TR, 7.8 ms; TE, minimum full; flip angle, 20°; matrix size, 256 × 256; 176 slices; voxel size, 1.13 × 1.13 × 1 mm). The 9 min resting-state fMRI data were acquired using a gradient single-shot 10 echo-planar imaging sequence (TE, minimum full; flip angle, 90°; matrix, 64 × 64; FOV, 192 × 192 mm; voxel size, 3 × 3 × 3 mm; TR, 3,000 ms; 60 slices with no gap).

##### Study 5 preprocessing of fMRI data

fMRI data were preprocessed using SPM12 (http://www.fil.ion.ucl.ac.uk/spm) and CONN (v.18b) (https://www.nitrc.org/projects/conn; Whitfield-Gabrieli and Nieto-Castanon, 2012) implemented in Matlab (R2018a; https://uk.mathworks.com/products/matlab). Preprocessing steps followed CONN's default pipeline and included motion estimation and correction by volume realignment using a six-parameter rigid body transformation, slice time correction, and simultaneous gray matter (GM), white matter (WM), and cerebrospinal fluid (CSF) segmentation and normalization to MNI152 stereotactic space (2 mm isotropic) of both functional and structural data. Following preprocessing, the following potential confounds were statistically controlled for six motion parameters calculated at the previous step and their first and second order derivatives, volumes with excessive movement (motion >0.5 mm and global signal changes larger than *z* = 3), linear drifts, and five principal components of the signal from WM and CSF (CompCor approach; Behzadi et al., 2007). Finally, data were bandpass filtered between 0.01 and 0.1 Hz. No global signal regression was performed (Vos de Wael et al., 2017).

### Analysis of the percentage of activated/deactivated voxels

To understand the role of subdivisions of DMN in semantic tasks, we extracted numbers of activating and deactivating voxels, relative to the implicit baseline, for each participant within three DMN subsystems—medial temporal (MT), core (Core), and frontotemporal (FT), in Studies 1–4. The networks of interest were defined using the Yeo et al. (2011) 17-network parcellation of 1,000 resting-state fMRI datasets projected into individual space. The three subsystems were mutually exclusive and did not overlap. The numbers of voxels in each of the three subsystems were as follows: 2,680 voxels within MT, 11,435 voxels within Core and 12,672 voxels within FT. The implicit baseline in all analyses consisted of periods of unmodeled time, for example, between blocks of trials, in which participants were not instructed to do anything. Although “resting periods” are not an ideal baseline for semantic activation (and deactivation), given that semantic cognition will continue (Binder et al., 1999), this approach provided an opportunity to compare responses with a common baseline.

In the analysis presented in the main body for each task-based study, individual activation maps were resampled to the voxel size of 2 × 2 × 2 mm^3^, aligned to the MNI 152 standard space, thresholded at *z* = 1.96, and binarized depending on whether the *z* value of each voxel passed the threshold. Then activating/deactivating voxels were represented as a percentage of over-threshold voxels in each subsystem. Analyses using different thresholds (*z* = 2.3; *z* = 2.6) showed similar effects (https://osf.io/vtuh4/). The advantage of this method is that it can reveal brain areas in which voxels both activate and deactivate in response to task demands; this pattern might correspond to a more specific response or connection pattern during a task which cannot be identified if activation levels are averaged across all the voxels within a region. The more standard univariate approach, averaging across voxels, is provided in an analysis for comparison on OSF (https://osf.io/vtuh4/).

The data from each study were examined separately using repeated-measures ANOVA, allowing us to make inferences about the roles of the DMN subsystems in internally oriented (recall) versus externally oriented (reading) states (Study 1), and in semantic tasks involving different input modalities (pictures/written words; Study 2), words that were abstract or concrete (Study 3), and following different types of semantic cues (emotion/location; Study 4). Our main analysis does not compare activation and deactivation between these four experiments directly, since the studies involved different scanners, acquisition protocols, and participants, had different spatial resolution and smoothing parameters, and employed tasks that lasted for different durations and that varied in difficulty. These differences were expected to affect metrics relating to DMN activation and deactivation; therefore, global differences between the studies (i.e., across conditions) were not expected to be fully interpretable in terms of cognitive processes. However, these aspects were well-matched within each study, allowing a series of functional inferences to be made relating to the functional contributions of each DMN subsystem. For each study, repeated-measures ANOVAs examined interactions of task condition by network for activated and deactivated voxels separately. These were followed by analyses of each network separately. When necessary, we also report post hoc Bonferroni-corrected *t* tests to characterize task interactions within networks. For each study, repeated-measures ANOVAs examined interactions of task condition by network for activated and deactivated voxels separately. These were followed by analyses of each network separately. When necessary, we also report post hoc Bonferroni-corrected *t* tests to characterize task interactions within networks. An analysis which uses linear mixed effects models to provide a direct comparison across datasets is available on OSF (https://osf.io/vtuh4/; “Comparisons_across_datasets.pdf”).

Despite the many differences between the experiments, we observed similarities in the locations of activating and deactivating voxels across experiments. To identify the typical location of activating and deactivating voxels, we added together the individual-level maps of all conditions across four studies and thresholding these combined maps at 20% percent of all datasets. All brain figures were created using BrainNet Viewer (http://www.nitrc.org/projects/bnv/; Xia et al., 2013).

### Functional connectivity of the commonly activated and deactivated DMN regions

**Figure 5.**
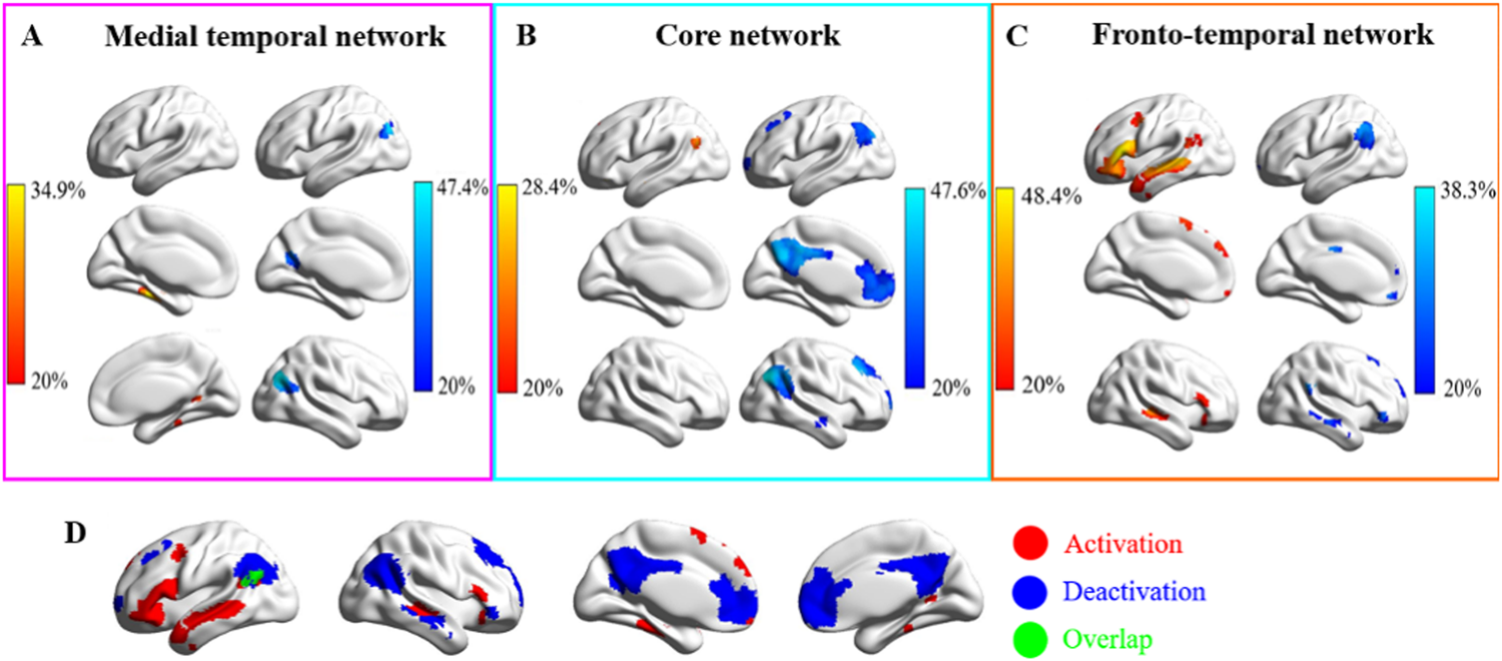
***A–C***, Regions that commonly activated or deactivated in the three subsystems across the four task-fMRI datasets. ***A–C***, Regions commonly activated or deactivated in MT, core, and FT subsystems, respectively. The values in the activation or deactivation maps represent the percentage of participants that activated or deactivated in the three subsystems (red, activation; blue, deactivation; green, overlap regions). ***D***, Activated and deactivated regions within the whole DMN (binarized from the maps from ***A–C***).

To identify the locations of commonly activated and deactivated voxels with the DMN subsystems, for each dataset: (1) we binarized each individual thresholded activation map and (2) added together these maps for each condition separately—so that for each condition, we had a map containing values within each voxel for the number of participants with over-threshold activation. We, then, (3) divided each of these maps by the number of participants in the respective dataset (i.e., for Studies 1–4, we divided the values in maps by 29, 22, 19, and 26, respectively) and then (4) applied a threshold of 20% for each condition in each dataset to locate the commonly activated and deactivated voxels within each subsystem (see panels C in Figs. 1–4). These steps 1–4 were applied separately for activating and deactivating voxels. In the final step, to locate the commonly activated and deactivated voxels across all studies within each DMN subsystem, we added the maps for all conditions across all datasets together and applied an overall threshold of 20% to the combined maps (Fig. 5*A*–*C*). These maps were then binarized to form the functional connectivity seeds (Fig. 5*D*).

In Study 5, we examined the intrinsic connectivity of the commonly activated and deactivated regions for each of the three subsystems (i.e., six seeds in total) in resting-state fMRI. In a first-level analysis, we computed whole-brain seed-to-voxel correlations for each seed in the same model after the BOLD time series were preprocessed and denoised. For the group-level analysis, we extracted seed-to-voxel functional connectivity at rest for 176 participants and performed contrasts between the functional connectivity maps from activating and deactivating seeds across the different subsystems (i.e., MT vs Core, FT vs Core, MT vs FT). Group-level analyses in CONN used a voxel threshold of *p* < 0.001 and were cluster-size FWE corrected at *p* < 0.05 (two-sided tests, Bonferroni corrected to *p* < 0.017 to account for the three contrasts). The functional connectivity maps for each contrast were uploaded to Neurovault (https://neurovault.org/collections/14749/; Gorgolewski et al., 2015) and decoded using Neurosynth (Yarkoni et al., 2011). The top 20 terms relevant to the maps were rendered as word clouds using a word cloud generator (https://www.wordclouds.com/).

### Gradient analysis of DMN connectivity patterns

To locate the intrinsic connectivity of the commonly activated and deactivated regions for each DMN subsystem within the topographical space defined by whole-brain cortical gradients, we computed the spatial correlations between our intrinsic connectivity maps and the top 3 cortical gradients defined by Margulies et al. (2016). The cortical gradients were extracted using diffusion embedding from a whole-brain connectivity matrix and represented components of spatial variance in intrinsic connectivity across the cortical surface (maps of Gradients 1–3 are from Margulies et al., 2016; https://neurovault.org/collections/1598/). Gradient 1 (the principal gradient, explaining the most variance) differentiates the connectivity patterns of heteromodal DMN (with positive values) and sensory-motor regions (with negative values). Gradient 2 captures the separation in connectivity between visual (positive) and auditory-motor (negative) connectivity patterns. Gradient 3 differentiates heteromodal regions that support more controlled cognition (positive) from core DMN (negative). These spatial correlations with each gradient were computed at the individual level, allowing us to perform repeated-measures ANOVAs that examined the location of activating and deactivating voxels for each DMN subsystem in gradient space.

## Results

### Analysis of the percentage of activated/deactivated voxels

#### Study 1: reading/autobiographical memory task

We extracted the percentage of activating and deactivating voxels during reading and autobiographical recall within each subsystem (Fig. 1). The conflict and no conflict conditions did not show any differences, so here we report repeated-measures ANOVAs including task (reading or recall) by DMN subsystem (MT, Core, or FT) for activated or deactivated voxels (models that include the conflict manipulation are available on OSF; https://osf.io/vtuh4/).

##### Activation

Activating voxels showed a main effect of task (*F*_(1,28)_ = 5.36; *p *= 0.028; *η*^2^*^ ^*= 0.16), with stronger activation for recall than for reading, a significant main effect of DMN subsystem (*F*_(2,56)_ = 22.14; *p *< 0.001; *η*^2^*^ ^*= 0.44), and a significant interaction effect between task and DMN subsystem (*F*_(2,56)_ = 22.18; *p *< 0.001; *η*^2^*^ ^*= 0.44). Paired *t* tests (corrected for the number of comparisons) compared the percentage of voxels activating across subsystems: the FT subsystem showed more activation than MT (*t*_(28)_ = 4.22; Bonferroni-corrected *p *< 0.001), while the core showed less activation than both MT (*t*_(28)_ = −2.56; Bonferroni-corrected *p *= 0.048) and FT subsystems (*t*_(28)_ = −5.88; Bonferroni-corrected *p *< 0.001). To understand the interaction between subnetwork and task, paired *t* tests compared reading and recall in each subsystem: in the MT and Core, reading elicited less activation than recall (MT: *t*_(28)_ = −3.45, Bonferroni-corrected *p *= 0.006; Core: *t*_(28)_ = −3.20, Bonferroni-corrected *p *= 0.009), while there was no significant difference in the FT subsystem (Bonferroni-corrected *p *= 0.249).

##### Deactivation

Deactivating voxels showed a significant main effect of task (*F*_(1,28)_ = 28.13; *p *< 0.001; *η*^2^*^ ^*= 0.50), with more deactivation for reading than that for recall, a significant main effect of DMN subsystem (*F*_(2,56)_ = 22.01; *p *< 0.001; *η*^2^*^ ^*= 0.44), and a significant interaction effect between task and DMN subsystem (*F*_(2,56)_ = 21.94; *p *< 0.001; *η*^2^*^ ^*= 0.44). The Core subsystem deactivated more than the MT and FT subsystems (Bonferroni-corrected *p *< 0.001), and there was no significant difference in deactivation between the MT and FT subsystems (Bonferroni-corrected *p *= 0.297). To understand the interaction effect, paired *t* tests were conducted between reading and recall in each subsystem. Reading elicited more deactivation than recall in the MT and Core subsystems (MT: *t*_(28)_ = 4.18, Bonferroni-corrected *p *< 0.001; Core: *t*_(28)_ = 5.57, Bonferroni-corrected *p *< 0.001), and there was no significant difference in the FT (FT: *t*_(28)_ = 2.11, Bonferroni-corrected *p *= 0.132).

##### Study 1 summary

The Core subsystem showed substantial task-related deactivation, especially during reading. The MT subsystem activated more and deactivated less during recall than that during reading. The FT subsystem was activated during both reading and recall and showed no differences between tasks. These results are consistent with the view that DMN subsystems have unique functional responses. Core DMN appears to be most decoupled from visual and attentional states (Zhang et al., 2022), even for tasks that involve semantic cognition and that are relatively naturalistic (i.e., reading as opposed to semantic decisions). The MT subsystem, in contrast, shows stronger activation to memory-based tasks, while the FT subsystem activates to both externally oriented (reading) and internally oriented (recall) tasks, consistent with the view that this DMN subsystem supports semantic cognition (Andrews-Hanna and Grilli, 2021). However, reading is a highly verbal task, while autobiographical memory is likely to involve more visuospatial processes that support internal scene construction (Andrews-Hanna et al., 2014; Andrews-Hanna and Grilli, 2021). To establish whether differences between the FT and MT subsystems reflect perceptually coupled versus decoupled aspects of cognition, or alternatively might reflect the recruitment of more abstract/verbal versus visuospatial codes, we compared the activation and deactivation of these subsystems across externally presented verbal and picture-based semantic decisions in Study 2.

#### Study 2: word/picture semantic judgment task

This task allowed us to compare activation and deactivation for semantic decisions involving words and pictures, which also varied in difficulty. The probe–target pairs were either strongly associated (easy trials) or weakly associated (hard trials). We extracted the percentage of activating and deactivating voxels in these four conditions: word-easy, word-hard, picture-easy, picture-hard, within each subsystem, and performed a 2 (modality: word or picture) by 2 (difficulty: easy or hard) by 3 (DMN subsystem: MT, Core, or FT) repeated-measures ANOVA for activating and deactivating voxels, respectively.

##### Activation

For activating voxels, there was no main effect of modality (*F*_(1,21)_ = 2.33; *p *= 0.14; *η*^2^*^ ^*= 0.10), a significant main effect of difficulty (*F*_(1,21)_ = 15.50; *p *< 0.001; *η*^2^*^ ^*= 0.43), with hard conditions activating more voxels than easy conditions, and a significant main effect of DMN subsystem (*F*_(1,21)_ = 33.31; *p *< 0.001; *η*^2^*^ ^*= 0.61), with the Core subsystem showing fewer activating voxels relative to MT (*t*_(21)_ = −5.66, Bonferroni-corrected *p *< 0.001) or FT (*t*_(21)_ = −14.61, Bonferroni-corrected *p *< 0.001), and no significant difference between the MT and FT (*t*_(21)_ = 0.60, Bonferroni-corrected *p *> 1). There were interaction effects between task modality and DMN subsystem (*F*_(1,21)_ = 73.32; *p *< 0.001; *η*^2^*^ ^*= 0.78), and between difficulty and DMN subsystem (*F*_(1,21)_ = 10.63; *p *< 0.001; *η*^2^*^ ^*= 0.34), but no other significant interactions.

To understand the significant two-way interactions for activation, we conducted 2 (modality: word or picture) by 2 (difficulty: easy or hard) repeated-measures ANOVAs for each subsystem. In MT, activating voxels showed a significant main effect of task modality (*F*_(1,21)_ = 38.33; *p *< 0.001; *η*^2^*^ ^*= 0.65), with picture conditions eliciting more activating voxels than word conditions; there was no main effect of difficulty (*F*_(1,21)_ = 3.33; *p *= 0.08; *η*^2^*^ ^*= 0.14) and no interaction effect between modality and difficulty (*F*_(1,21)_ = 2.26; *p *= 0.15; *η*^2^*^ ^*= 0.10). In the Core subsystem, activating voxels showed no main effect of modality (*F*_(1,21)_ = 1.09; *p *= 0.31; *η*^2^*^ ^*= 0.05), a near significant main effect of difficulty (*F*_(1,21)_ = 3.91; *p *= 0.061; *η*^2^*^ ^*= 0.16) with hard conditions activating marginally more voxels than easy conditions, and no significant interaction effect (*F*_(1,21)_ = 3.06; *p *= 0.095; *η*^2^*^ ^*= 0.13). In the FT, activating voxels showed a main effect of modality (*F*_(1,21)_ = 119.29; *p *< 0.001; *η*^2^*^ ^*= 0.85), with verbal conditions eliciting more activation than pictures, a significant main effect of difficulty (*F*_(1,21)_ = 35.69; *p *< 0.001; *η*^2^*^ ^*= 0.63), with hard conditions activating more voxels than easy conditions, and no significant interaction effect (*F*_(1,21)_ = 0.10; *p *= 0.76; *η*^2^*^ ^*= 0.005).

##### Deactivation

Deactivating voxels showed a main effect of modality (*F*_(1,21)_ = 4.70; *p *= 0.042; *η*^2^*^ ^*= 0.18), with picture conditions eliciting more deactivation than word conditions, and a significant main effect of difficulty (*F*_(1,21)_ = 25.73; *p *<0.001; *η*^2^*^ ^*= 0.55), with hard conditions eliciting more deactivation than easy conditions. There was also a main effect of DMN subsystem (*F*_(1,21)_ = 122.75; *p *< 0.001; *η*^2^*^ ^*= 0.85), with the Core showing more deactivation relative to MT (*t*_(21)_ = 11.05; Bonferroni-corrected *p *< 0.001) or FT (*t*_(21)_ = 14.19; Bonferroni-corrected *p *< 0.001) and MT showing more deactivation relative to FT (*t*_(21)_ = 3.54, Bonferroni-corrected *p *= 0.006). There were interactions between modality and subsystem (*F*_(1,21)_ = 33.14; *p *< 0.001; *η*^2^*^ ^*= 0.61) and difficulty and subsystem (*F*_(1,21)_ = 9.18; *p *< 0.001; *η*^2^*^ ^*= 0.30), but no other significant interactions.

To understand the significant two-way interactions for deactivation, we conducted 2 (modality: word or picture) by 2 (difficulty: easy or hard) repeated-measures ANOVAs for each subsystem. MT showed a main effect of modality (*F*_(1,21)_ = 6.43; *p *= 0.019; *η*^2^*^ ^*= 0.23), with words eliciting more deactivation than pictures, a main effect of difficulty (*F*_(1,21)_ = 28.49; *p *< 0.001; *η*^2^*^ ^*= 0.58), with hard conditions eliciting more deactivation than easy conditions, and no significant interaction (*F*_(1,21)_ = 1.90; *p *= 0.18; *η*^2^*^ ^*= 0.08). Core DMN showed a main effect of task modality (*F*_(1,21)_ = 8.26; *p *= 0.009; *η*^2^*^ ^*= 0.28), with pictures eliciting more deactivation than words, a main effect of difficulty (*F*_(1,21)_ = 20.30; *p *< 0.001; *η*^2^*^ ^*= 0.49), with more deactivation for hard than easy conditions, and no significant interaction (*F*_(1,21)_ = 0.37; *p *= 0.55; *η*^2^*^ ^*= 0.02). FT showed the opposite effect of modality (*F*_(1,21)_ = 31.33; *p *< 0.001; *η*^2^*^ ^*= 0.60), with more deactivation for pictures than for words, a main effect of difficulty (*F*_(1,21)_ = 5.37; *p *= 0.031; *η*^2^*^ ^*= 0.20), with more deactivation for hard than easy conditions, and no interaction (*F*_(1,21)_ = 0.00; *p *= 0.99; *η*^2^*^ ^*= 0.00).

##### Study 2 summary

The MT subsystem showed more activation for pictures and more deactivation for words, while FT showed the opposite pattern. Core DMN showed little activation for either words or pictures, yet more deactivation for pictures, broadly consistent with the proposal that this network shows perceptual decoupling (Andrews-Hanna et al., 2014). The DMN subsystems also showed different responses to the difficulty manipulation: while all three networks showed more deactivation in response to harder judgments, in line with the “task-negative” expectation for DMN regions (Raichle et al., 2001; Greicius et al., 2003), the FT subsystem also showed significantly more activation for difficult semantic trials. FT shows both activation and deactivation in response to harder semantic decisions, suggesting it shows more specific patterns of response under demanding circumstances, a pattern that we refer to as “tuning.”

These results help to constrain interpretations of functional distinctions between DMN subsystems. We found that MT is not only implicated in autobiographical memory but also in external tasks involving pictures as opposed to words, suggesting it supports visuospatial representations across both external and internal modes of cognition. In contrast, the FT subsystem is more strongly implicated in verbal semantic tasks, suggesting that this network might support abstract aspects of semantic processing. The next study tests this proposal directly by comparing responses with semantic decisions involving concrete and abstract words.

#### Study 3: abstract/concrete word synonym judgment task

We extracted the percentage of activating and deactivating voxels during semantic decisions involving abstract and concrete words within each subsystem and performed a 2 (task: abstract or concrete) by 3 (DMN subsystem: MT, Core, or FT) repeated-measures ANOVA examining activation and deactivation voxels, respectively. There were no significant effects of cueing; therefore, this experimental factor is omitted below (see full analysis on OSF; https://osf.io/vtuh4/).

##### Activation

For activating voxels, there was no main effect of concreteness (*F*_(1,18)_ = 0.10; *p *= 0.75; *η*^2^*^ ^*= 0.006), a significant main effect of DMN (*F*_(2,36)_ = 30.02; *p *< 0.001; *η*^2^*^ ^*= 0.63), and a significant interaction effect between concreteness and DMN subsystem (*F*_(2,36)_ = 13.45; *p *< 0.001; *η*^2^*^ ^*= 0.43). FT activated more than MT (*t*_(18)_ = 5.66; Bonferroni-corrected *p *< 0.001) and Core (*t*_(18)_ = 6.80, Bonferroni-corrected *p *< 0.001), while the difference in activation between MT and Core was not significant (*t*_(18)_ = 2.08; Bonferroni-corrected *p *= 0.159). To understand the interaction effect, paired *t* tests were conducted between abstract and concrete words in each subsystem: there was no effect of this manipulation in either the MT or Core subsystems (MT: *t*_(18)_ = 1.78, Bonferroni-corrected *p *= 0.276; Core: *t*_(18)_ = 1.22, Bonferroni-corrected *p *= 0.711), while abstract words elicited more activation than concrete words in the FT subsystem (*t*_(18)_ = 3.80; Bonferroni-corrected *p *= 0.003).

##### Deactivation

For deactivating voxels, there were main effects of concreteness (*F*_(1,18)_ = 59.94; *p *< 0.001; *η*^2^*^ ^*= 0.77), and DMN subsystem (*F*_(2,36)_ = 20.73; *p *< 0.001; *η*^2^*^ ^*= 0.54), plus an interaction between them (*F*_(2,36)_ = 5.86; *p *= 0.018; *η*^2^*^ ^*= 0.25). FT deactivated less than MT (*t*_(18)_ = 5.40; Bonferroni-corrected *p *< 0.001) and Core subsystems (*t*_(18)_ = 6.79; Bonferroni-corrected *p *< 0.001), while the MT and the Core subsystems did not differ (*t*_(18)_ = 1.63; Bonferroni-corrected *p *= 0.36). Paired *t* tests to examine the interaction showed that abstract words elicited more deactivation than concrete words in all three subsystems (MT: *t*_(18)_ = 4.68, Bonferroni-corrected *p *< 0.001; Core: *t*_(18)_ = 9.17, Bonferroni-corrected *p *< 0.001; FT: *t*_(18)_ = 5.54, Bonferroni-corrected *p *< 0.001), but this effect was largest in the Core.

##### Study 3 summary

The FT subsystem showed stronger activation to abstract than concrete concepts, consistent with the interpretation that it supports abstract aspects of meaning. FT DMN regions, for example, in lateral anterior temporal lobes, are thought to lie at the end of a processing stream that supports the abstraction of meaning from diverse sensory-motor features. Yet the FT subsystem also showed greater deactivation for abstract concepts (i.e., a tuning effect, like the effect of difficulty found for FT in Study 2). Abstract concepts along with more difficult semantic decisions might require a more selective pattern of DMN activation and connectivity. The MT subsystem was equally activated by abstract and concrete conditions, and both MT and Core DMN deactivated more to abstract words. Core DMN also appeared to show more activating voxels in this task, in which verbal semantic decisions were made following a meaningful sentence cue, as opposed to Study 2, in which semantic decisions occurred in the absence of a context.

The evidence presented so far demonstrates greater activation for verbal semantic tasks and abstract concepts in FT DMN, and for picture semantic tasks and autobiographical memory retrieval in MT DMN, although we also found that FT DMN responds in a similar way to internally oriented and externally oriented tasks that involve meaning (Study 1). Abstract words also entail affective processing to a greater extent than concrete concepts (Kousta et al., 2011; Vigliocco et al., 2014). To clarify the effect of emotion in driving differences in DMN function, the next study compared activation for facial portrayals of emotion and pictures of spatial locations in the three subsystems.

#### Study 4: emotional/spatial cues task

We extracted the percentage of activating and deactivating voxels within each subsystem during semantic decisions that followed emotion cues (facial portrayals of emotion) and visuospatial cues (photographs of scenes) and performed a 2 (cue type: emotion or location) by 3 (DMN subsystem: MT, Core, or FT) repeated-measures ANOVA for the percentage of activated and deactivated voxels, respectively. An additional analysis examined the effect of multiple cues on DMN recruitment (the two-cue condition that leveraged both location and emotion cues within a trial; since this analysis does not reveal effects of emotion vs scenes, these results are available on OSF; https://osf.io/vtuh4/).

##### Activation

Activation showed a main effect of cue type (*F*_(1,25)_ = 7.64; *p *= 0.011; *η*^2^*^ ^*= 0.23) with spatial cues activating more voxels than emotional cues, a main effect of DMN (*F*_(2,50)_ = 13.24; *p *< 0.001; *η*^2^*^ ^*= 0.35), and a significant interaction effect between cue type and DMN subsystem (*F*_(2,50)_ = 26.76; *p *< 0.001; *η*^2^*^ ^*= 0.52). The MT and FT activated more than the Core (*t*_(25)_ = 4.39, Bonferroni-corrected *p *< 0.001 for the MT; *t*_(25)_ = 3.35, Bonferroni-corrected *p *= 0.009 for the FT), while the MT and FT subsystems did not differ (*t_(_*_25)_ = 1.96; Bonferroni-corrected *p *= 0.183). To understand the interaction effect, paired *t* tests compared the response to emotion and spatial cues in each subsystem: emotional cues elicited less activation than spatial cues in MT (*t*_(25)_ = 5.07; Bonferroni-corrected *p *< 0.001), while there was no significant difference either in the Core or FT subsystems (Bonferroni-corrected *p *> 1).

##### Deactivation

Deactivating voxels showed no main effect of cue type (*F*_(1,25)_ = 0.025; *p *= 0.88; *η*^2^*^ ^*= 0.001), a main effect of DMN subsystem (*F*_(2,50)_ = 9.11; *p *< 0.001; *η*^2^*^ ^*= 0.27), and a significant interaction (*F*_(2,50)_ = 3.90; *p *= 0.027; *η*^2^*^ ^*= 0.14). MT and FT deactivated less than Core DMN (*t*_(25)_ = 4.84, Bonferroni-corrected *p *< 0.001 for MT; *t*_(25)_ = 3.46, Bonferroni-corrected *p *= 0.006 for FT), while the MT and FT subsystems did not differ (*t*_(25)_ = 1.15; Bonferroni-corrected *p *= 0.786). Paired *t* tests compared deactivation in response to emotion and spatial cues for each subsystem but no significant differences were found (*t*_(25)_ < 2.07; Bonferroni-corrected *p *> 0.147).

##### Study 4 summary

The MT subsystem activated significantly more to spatial than emotional cue conditions. The Core subsystem deactivated to both conditions without a significant difference between them. The FT subsystem activated to both conditions equally, confirming that different kinds of meaningful features can activate this network. These results suggest that even though valence is thought to be a crucial component of the meaning of abstract words (Kousta et al., 2011; Vigliocco et al., 2014), a simple response to valence is unlikely to account for the role of the FT subsystem in abstract semantics. The results also provide a further demonstration that activation in the MT subsystem relates to visuospatial coding.

### Regions activated and deactivated in DMN subsystems

The subsystems of DMN showed different functional responses across a set of semantic tasks. The FT subsystem showed an increase in the number of activated voxels (relative to the implicit baseline) for both reading and autobiographical memory and showed a stronger response to words and abstract concepts; this suggests it supports semantic tasks that involve abstract concepts across both perceptually coupled and decoupled tasks. The MT subsystem showed an increase in activated voxels (relative to the implicit baseline) for autobiographical memory and pictorial semantic judgments and an increase in deactivated voxels for abstract concepts, suggesting that a visuospatial code is core to its behavior, although scenes do not need to be generated internally. The Core subsystem showed an increased in deactivated voxels (relative to the implicit baseline) in response to most of the externally oriented tasks but voxels were both activated and deactivated during autobiographical memory, in line with the view that this subsystem supports perceptually decoupled cognition.

We next considered where these regions of activating and deactivating voxels were located within each subsystem across participants and tasks. This is shown in the bottom panels of Figures 1–4. The MT subnetwork showed activating voxels, relative to baseline, in medial temporal regions (particularly for autobiographical memory retrieval in Study 1 and across verbal and picture associations in Study 2). The angular gyrus (AG) subcomponent of MT showed more deactivating voxels in Studies 1 and 2, when people accessed nonspatial meanings from inputs, especially in the right hemisphere (in Studies 1 and 3). However, both medial temporal and inferior parietal components of this network contained activating voxels when people made semantic decisions that were cued by images of scenes in Study 4. Across studies, the MT subsystem reliably showed activating voxels in bilateral medial temporal regions and commonly deactivating voxels in AG and medial occipitoparietal cortex (Fig. 5*A*).

Core DMN frequently showed deactivation. All nodes of this network showed deactivating voxels during demanding semantic decisions to words and pictures (Study 2), and most showed overlapping areas of activating and deactivating voxels during semantic decisions that followed sentences which were sometimes related in meaning (Study 3). However, activating voxels were seen in left AG when people retrieved autobiographical memories (Study 1) and when they made semantic decisions following previously presented faces and scenes that disambiguated the meaning of words (Study 4). Across studies, the Core subsystem showed activating voxels in common areas of left AG, and deactivating voxels in more dorsal and posterior bilateral AG, posterior cingulate cortex, superior frontal gyrus, and frontal pole (Fig. 5*B*).

The FT subsystem showed activating voxels (relative to the implicit baseline) in left hemisphere regions within the semantic network (left inferior frontal gyrus, anterior temporal cortex, and AG) in Studies 1 and 4 involving reading, autobiographical memory, and word judgments following face and location cues. In these studies, there were more deactivating voxels in the right hemisphere. For very demanding semantic decisions in Studies 2 and 3, a somewhat different pattern was seen. There were more activating voxels bilaterally (or overlapping areas of activating and deactivating voxels) in inferior frontal gyrus (IFG) and a similar response in anterior temporal cortex across hemispheres (although more deactivation in Study 2 and more activation in Study 3). Across studies, the FT subsystem showed activating voxels in left inferior frontal gyrus (IFG), left AG, and bilateral superior temporal gyrus (STG) and deactivating voxels in other regions of left AG as well as right IFG and STG (Fig. 5*C*).

When combining data across all subsystems (Fig. 5), there were reliable regions showing activating voxels across participants and tasks in regions associated with semantic processing, including left temporal cortex, and left inferior frontal gyrus, along with medial temporal cortex. There were common areas containing deactivating voxels in the midline anterior and posterior cingulate cortex, broad swathes of the right AG, and specific areas of the left AG (We also examined spatial correlations between the patterns of activation and deactivation across studies that were used to drive intrinsic connectivity analysis and the response pattern for each condition separately, within the DMN. Spatial correlations were high for most conditions (averaging 0.57 across 8 tasks for activation, and 0.76 for deactivation; see www.osf.io/vtuh4/)). The left AG was the site that most reliably showed both activating and deactivating voxels, which might relate to response “tuning,” as specific task-irrelevant representations or connectivity patterns are suppressed (see Studies 2 and 3). In this way, we replicated and extended some previous observations showing the left anterior temporal cortex activates while the AG deactivates in semantic tasks (Humphreys et al., 2015); however, the AG also shows activation in some studies, consistent with our findings of mixed responses, both above and below baseline, at this site (Murphy et al., 2018; Kuhnke et al., 2023).

### Functional connectivity of the commonly activated and deactivated DMN regions

**Figure 6.**
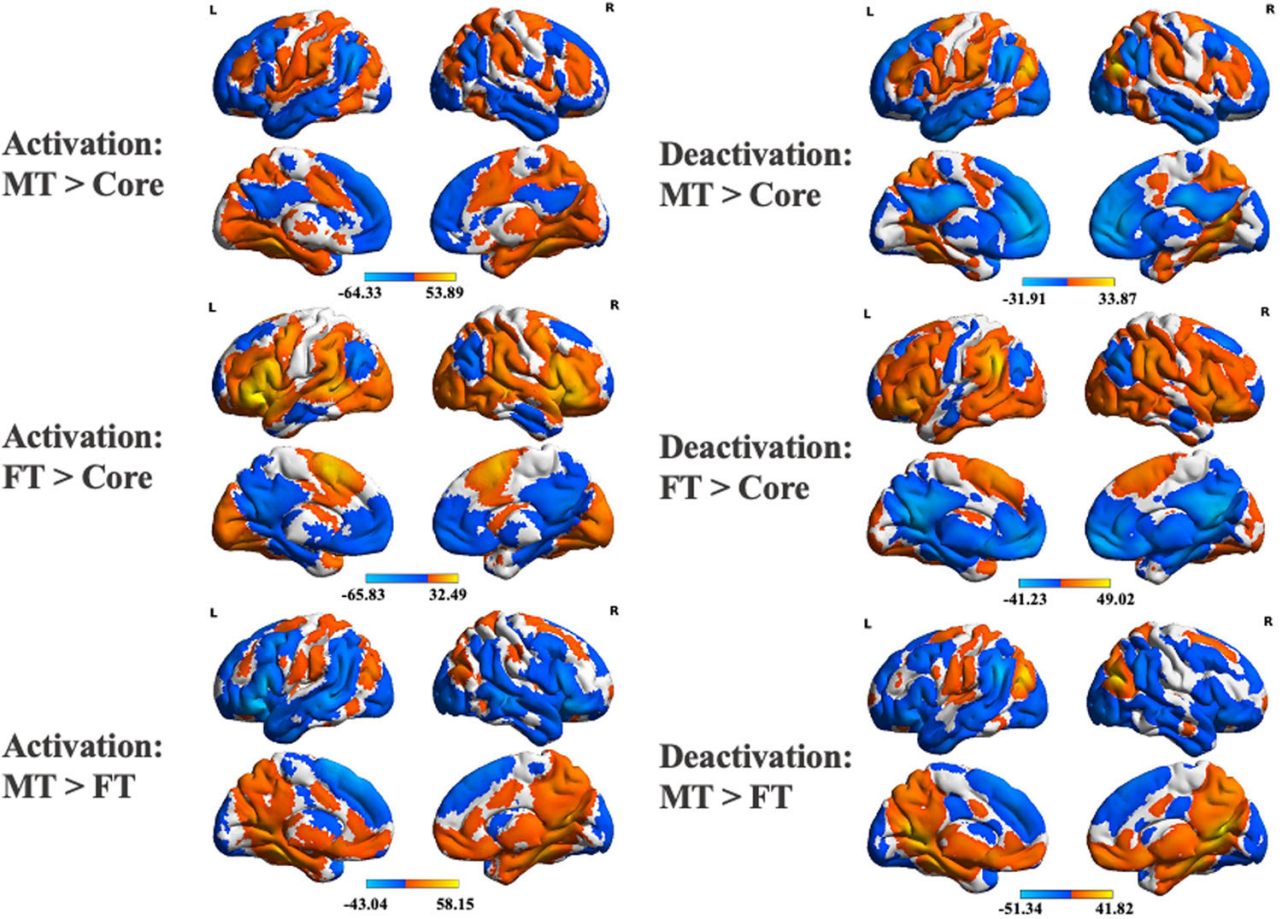
Contrasts between functional connectivity maps seeding from commonly activated regions in the three subsystems. Each contrast map was FWE-corrected to the voxel-level *p* < 0.001 and cluster-level *p* < 0.05. ***A–C***, Contrasts of MT versus Core, FT versus Core, and MT versus FT, respectively. The left and right panels correspond to the maps for activation and deactivation seeds, respectively.

To understand how areas of activation and deactivation in each DMN subnetwork are functionally connected with the whole brain, we examined functional connectivity at rest. Basic contrasts between the functional connectivity maps for activating and deactivating regions of each subsystem are shown in Figure 6. There was stronger connectivity to visual and motor regions for MT and FT than for Core, consistent with the proposal that the Core DMN is more perceptually decoupled. In addition, there was stronger connectivity to areas of dorsolateral prefrontal cortex and to anterior portions of inferior parietal cortex for MT and FT compared with Core DMN. The reverse contrasts, relating to stronger connectivity for Core DMN compared with MT and FT, highlighted regions of AG, posterior cingulate cortex, and medial prefrontal cortex associated with Core DMN, as well as areas of MT DMN when considering Core over FT, and areas of FT DMN when considering Core over MT. Direct contrasts of MT and FT DMN revealed stronger coupling of MT to the posterior cingulate, ventromedial prefrontal cortex, posterior parts of inferior parietal cortex, and motor cortex, while FT was more coupled to occipital pole, more anterior parts of AG, and swathes of ventrolateral and dorsolateral and dorsomedial prefrontal cortex as well as posterior middle temporal gyrus. All these effects were similar across seeds derived from activated and deactivated voxels for each network. In cognitive decoding of these connectivity maps, Core DMN was more associated with terms linked to episodic memory and mentalizing, MT was associated with terms such as “navigation,” and FT was associated with language terms.

**Figure 7.**
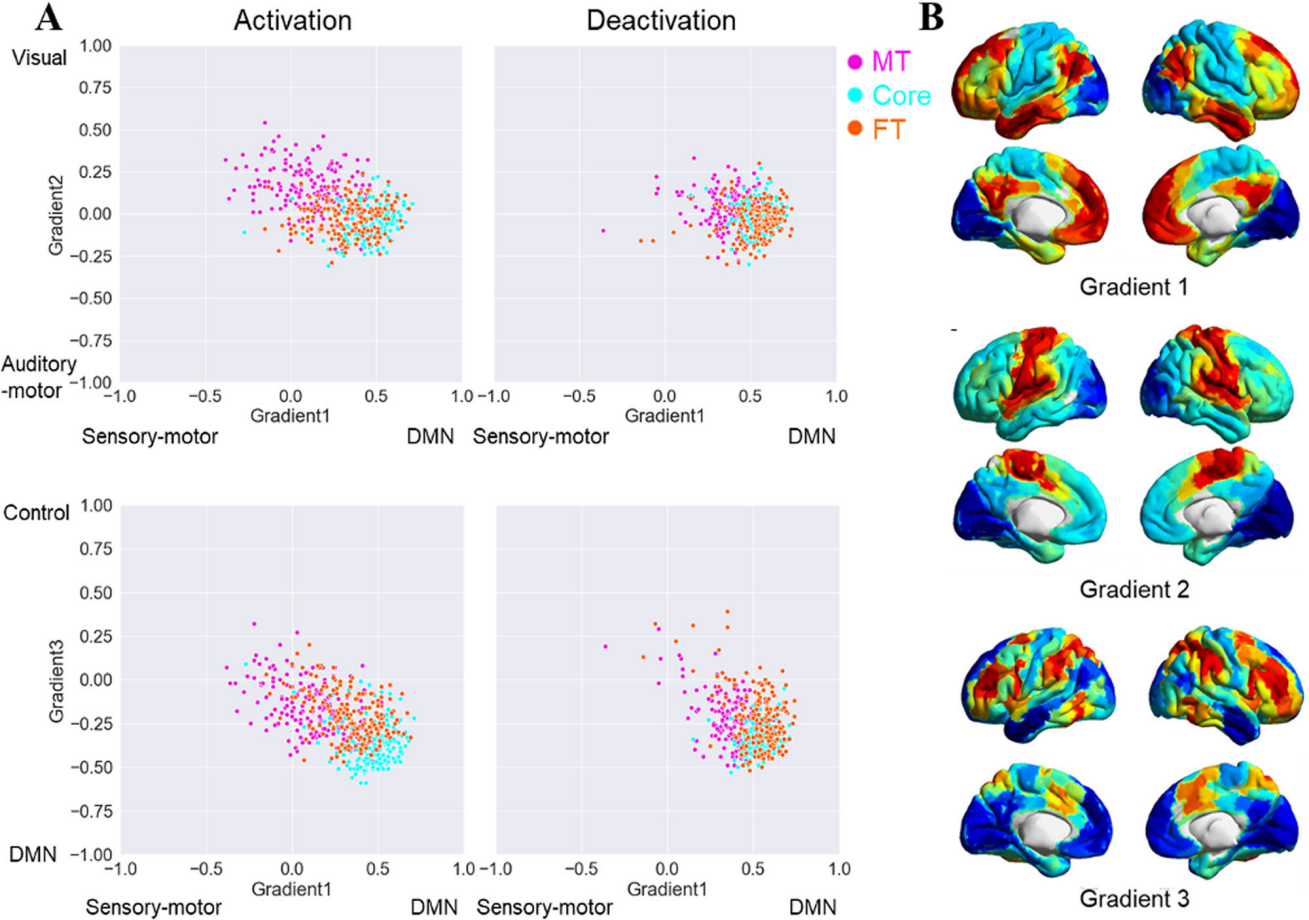
Scatter plots of the individual-level correlations between the functional connectivity maps seeding from commonly activated and deactivated regions in the three subsystems and the three cortical gradients (Fig. 7*A*, Margulies et al., 2016) and the maps of gradient one to three (Fig. 7*B*, taken from Shao et al., 2022).

In a final analysis, we situated the functional connectivity maps of commonly activated and deactivated DMN regions in the topographical space defined by whole-brain connectivity gradients (Margulies et al., 2016; Fig. 7). These describe dimensions of intrinsic connectivity that explain the most variance. By correlating DMN connectivity patterns for each subsystem with these gradients, we can establish dimensional differences between them that we expect to relate to the functional differences observed in Studies 1–4. Lower spatial correlations with Gradient 1 suggest functional responses that have greater similarity with unimodal regions, while higher correlations suggest more heteromodal and/or abstract responses. Lower spatial correlations with Gradient 2 indicate a stronger motor response, while higher spatial correlations indicate a stronger visual response. Higher spatial correlations with Gradient 3 suggest a response closer to control regions while lower correlations indicate greater similarity with heteromodal regions not associated with control.

For Gradient 1, we found a significant main effect of voxel activation (*F*_(1,175)_ = 725.63; *p *< 0.001; *η*^2^*^ ^*= 0.81), with higher positive (heteromodal/abstract-anchored) correlations for deactivating than activating voxels. There was a significant main effect of DMN subsystem (*F*_(2,350)_ = 310.91; *p *< 0.001; *η*^2^*^ ^*= 0.64); Core DMN showed a higher spatial correlation with Gradient 1 than both MT (Bonferroni-corrected *p *< 0.001) and FT (Bonferroni-corrected *p *< 0.001), while FT showed higher correlation than MT (Bonferroni-corrected *p *< 0.001). There was also a significant interaction between activation and subsystem (*F*_(2,350)_ = 52.94; *p *< 0.001; *η*^2^*^ ^*= 0.23). Gradient 1 correlations were higher for deactivation than those for activation for MT (*t*_(175)_ = 16.85; Bonferroni-corrected *p *< 0.001), FT (*t_(_*_175)_ = 15.50; Bonferroni-corrected *p *< 0.001), and Core (*t*_(175)_ = 8.66; Bonferroni-corrected *p *< 0.001), but this difference between activation and deactivation was smaller for core DMN.

For Gradient 2, we found a main effect of voxel activation (*F*_(1,175)_ = 42.87; *p *< 0.001; *η*^2^*^ ^*= 0.20) with higher spatial correlations (more visual response) seen for regions of activating than deactivating voxels. There was also a main effect of DMN subsystem (*F*_(2,350)_ = 205.89; *p *< 0.001; *η*^2^*^ ^*= 0.54): MT showed higher spatial correlation with the visual end of Gradient 2 than Core DMN (Bonferroni-corrected *p *< 0.001) or FT (Bonferroni-corrected *p *< 0.001), consistent with stronger visual representation within this subsystem. There was no difference in Gradient 2 values between the Core and FT (Bonferroni-corrected *p *= 1). Finally, Gradient 2 showed a significant interaction between activation and subsystem (*F*_(2,350)_ = 96.66; *p *< 0.001; *η*^2^*^ ^*= 0.36). Activation regions showed higher correlations with the visual end of Gradient 2 than deactivation regions for MT (*t*_(175)_ = 11.33, Bonferroni-corrected *p *< 0.001), while the Core showed the opposite pattern (*t*_(175)_ = 4.47, Bonferroni-corrected *p *< 0.001). There was no difference in Gradient 2 correlations across activation and deactivation for FT (*t*_(175)_ = 1.98, Bonferroni-corrected *p *= 0.147).

For Gradient 3, there was a main effect of voxel activation (*F*_(1,175)_ = 13.28; *p *< 0.001; *η*^2^*^ ^*= 0.07) reflecting higher correlations with the executive end of this gradient for activating than deactivating voxel regions. There was also a significant main effect of DMN subsystems (*F*_(2,350)_ = 147.03; *p *< 0.001; *η*^2^*^ ^*= 0.46): Core DMN showed lower correlations (less executive response) than MT (Bonferroni-corrected *p *< 0.001) and FT (Bonferroni-corrected *p *< 0.001), with no difference between MT and FT (Bonferroni-corrected *p *= 0.35). Gradient 3 also showed an interaction between activation and subsystem (*F*_(2,350)_ = 16.94; *p *< 0.001; *η*^2^*^ ^*= 0.09). Activating regions showed higher correlations (higher executive response) than deactivating regions for MT (*t*_(175)_ = 5.71; Bonferroni-corrected *p *< 0.001), while the Core showed the opposite pattern (less executive response; *t*_(175)_ = 3.71; Bonferroni-corrected *p *< 0.001). There was no difference between activating and deactivating voxels within FT (*t*_(175)_ = 1.75; Bonferroni-corrected *p *= 0.246).

These results show that deactivating voxels are closer to the heteromodal end of Gradient 1 than activating voxels, especially for the MT and FT subsystems, in line with the view that task-related activation of these subsystems is often driven by sensory inputs. We also found that activating voxels for the MT subsystem were more visual than deactivating voxels on Gradient 2, while the reverse was true of Core DMN (and no difference for FT). This might reflect the key importance of visual codes in the MT subsystem and perceptual decoupling in Core DMN. Finally, activating voxels in the MT subsystem were closer to the controlled end of Gradient 3 than deactivating voxels, while the reverse was found for Core DMN (again, no difference for FT).

## Discussion

By applying a novel method that examines both activating and deactivating voxels, our study reveals the contribution of DMN subsystems to semantic cognition, and how this is influenced by perceptual decoupling, input modality, abstractness, and spatial versus emotional features. None of the DMN variants were exclusively task negative. Instead, the recruitment of DMN subsystems varied according to the need to maintain information in memory that differs from inputs in the external world and the requirement to represent visuospatial and abstract conceptual information. Although DMN can be characterized as a unitary whole (Raichle et al., 2001; Yeo et al., 2011), the three subsystems played distinct and complementary roles in semantic cognition which were related to their different locations on a multidimensional space defined by whole-brain gradients of connectivity. Core DMN showed both activating and deactivating voxels during autobiographical memory and when semantic retrieval followed the presentation of earlier information maintained in working memory. It showed almost exclusive voxel deactivation when new trials were presented in the absence of a meaning-based context; this subsystem was also relatively far from unimodal, visual, and control regions in gradient space. MT was recruited in internally oriented tasks involving visuospatial imagery (autobiographical memory) and by pictorial semantic tasks; it was closer than core DMN to visual and control regions. FT showed more voxel activation for abstract verbal semantic processing and overlapping regions of task activation and deactivation; both activating and deactivating voxels were more numerous during more demanding semantic tasks, suggesting that semantic responses or patterns of cortical connectivity in this subsystem are “tuned” in the face of higher semantic control demands.

Our results align well with a recent topographic account suggesting that the diverse roles of the DMN relate to its spatial location on the cortical mantle (Margulies et al., 2016; Smallwood et al., 2021). In general, this network is distant from the sensory-motor cortex on the principal gradient, and this position is suited to cognition that is perceptually decoupled from the changing external environment or that builds on previously presented information (e.g., when decisions follow sentence cues): we observed this type of response in core DMN. However, this position on the principal gradient also places the DMN at the end of a processing stream (or streams) that allows the integration of features, giving rise to heteromodal representations that support cognition that is both guided by memory and driven by new sensory inputs: we observed this type of response in FT and MT DMN.

Our analysis of activation and deactivation in each task relied on a comparison with an implicit baseline in each study, in which participants were not engaged in time-locked aspects of the tasks. Previous research has shown that DMN regions can be more active during “resting” baseline periods, including implicit baselines, than those during tasks and there are several potential explanations of this pattern. First, it might reflect higher engagement of semantic representations and/or self-referential processes during mind-wandering which occurs during rest periods, compared with semantic tasks that require more focused patterns of information retrieval (Binder et al., 1999, 2009). In addition, given that DMN sits at the top of a unimodal–transmodal cortical hierarchy, deactivation during tasks might reflect functional “tuning” that suppresses inputs that are not task relevant (Amedi et al., 2005; Azulay et al., 2009). These accounts are not mutually exclusive, and we are unable to separate them empirically in the current study. Nevertheless, we show that the three DMN subsystems differ in terms of the extent to which they show this pattern of task-induced deactivation, with the core DMN almost always showing deactivation relative to baseline and FT DMN more commonly showing activation.

The responses we observed for core DMN are broadly in line with a “perceptual decoupling” view of the DMN and not with a “task-negative” view (Raichle et al., 2001; Greicius et al., 2003). Although core DMN showed more deactivation than the MT and FT subsystems across multiple semantic tasks, demonstrating that this subnetwork decouples from visual and attentional states (even during long-term memory retrieval in some circumstances), the numbers of activating and deactivating voxels were more similar when the task involved sustaining meaningful information over time (especially in the cued synonym judgment task in Study 3, but also to a lesser extent, in the autobiographical memory task in Study 1 and the emotion cue task in Study 4). The ability of core DMN to support conceptual information that is distinct from the unfolding sensory environment might be supported by its greater distance from unimodal and visual ends of the gradients compared with other subnetworks. The functional importance of this distance from visual cortex is also supported by our finding that activating voxels in core DMN were, on average, more distant from visual cortex than deactivating voxels. Moreover, the observation that core DMN voxels can activate over baseline during the maintenance of contextual information as well as during autobiographical memory retrieval is corroborated by recent studies showing core DMN responds when decisions are guided by working memory (Murphy et al., 2018, 2019), as well as showing stronger responses during autobiographical memory when participants report being more focused on the task and not distracted by concurrent visual input (Zhang et al., 2022). These findings indicate that even though core DMN contained more deactivating than activating voxels in the relatively internal autobiographical memory task, this network is not task-negative; instead, rest states providing the task baseline might promote mind-wandering states in which attention is directed toward internally maintained information (Christoff et al., 2009).

The MT subsystem showed stronger activation for autobiographical memory, pictorial semantic judgments, and semantic decisions following scene cues: in particular, it showed more activating than deactivating voxels when location photographs were presented as part of the task (in Study 4) and during autobiographical memory (in Study 1). These findings are consistent with the view that this subsystem is recruited when integrating visuospatial information and contributes to scene construction (Andrews-Hanna and Grilli, 2021). The MT subsystem reliably showed activation in bilateral medial temporal regions, consistent with previous studies showing these regions are important for thinking about events that happened in a specific place and time during episodic retrieval (Hassabis and Maguire, 2007; Hassabis et al., 2007). Moreover, intrinsic connectivity of activated regions within MT revealed stronger coupling with the visual end of the second gradient than other DMN subsystems. The role of MT in visuospatial tasks might be enhanced by this subsystem's relative proximity to unimodal and visual ends of the gradients, given that activating voxels were closer to visual cortex than deactivating voxels, in a reversal of the pattern for core DMN. Therefore, although core and MT subsystems are both implicated in episodic, internal states such as autobiographical memory retrieval (Andrews-Hanna, 2012), task activation in these subnetworks has opposite relationships with visual coupling.

The FT subsystem showed stronger activation for words than pictures and for abstract than concrete words, yet equivalent responses for reading and autobiographical memory, and for semantic decisions following location and emotion cues, suggesting a role in abstract conceptual processing, irrespective of semantic content or whether tasks are internally or externally oriented. It showed a larger number of activating than deactivating voxels in most of the tasks we examined, including reading, autobiographical memory, and in all the tasks employing semantic judgments to written words. However, it showed particularly strong responses to abstract and concrete synonym judgments presented after sentence cues in Study 3, consistent with the suggestion that this subsystem is more strongly engaged by verbal semantic processing in temporally extended contexts. Activating voxels in FT were situated between MT and Core on the heteromodal-to-unimodal gradient, in line with this subsystem's recruitment in both externally driven abstract semantic tasks and internal tasks such as autobiographical memory. Task-induced increases in activation and deactivation were also stronger for more demanding semantic judgments, suggesting that semantic representations are maintained in a “tuned” state when task demands are higher and suggesting that deactivation is not task irrelevant; instead, deactivation could suppress irrelevant features, input modalities, and patterns of connectivity (Schwartz et al., 2007; Gouws et al., 2014; Stiernman et al., 2021) or improve the processing efficiency and/or reduce the physiological cost of task responses (Szinte and Knapen, 2020). The left AG within the FT subsystem showed the most reliable activation and deactivation overlap across tasks, indicating the sensitivity of this site to changing task demands: this region may support diverse tasks by flexibly gating its connections, consistent with previous evidence showing it contains “echoes” of many other networks and dynamically modulates its response to suit the context (Braga et al., 2013; Spreng et al., 2013; Vatansever et al., 2015; Dixon et al., 2017, 2018; Wang et al., 2021).

Interestingly, we observed that the presentation of irrelevant information (effects of conflict in Study 1 and irrelevant cues in Study 3) did not modulate the response of the DMN subsystems, while the difficulty of semantic retrieval (effects of association strength in Study 2 and abstractness in Study 3) generated both increases and decreases in activation in FT. This corroborates the growing view that DMN deactivation cannot be distilled down to difficulty but is more likely to reflect selective integration of relevant semantic information (Smallwood et al., 2021), which is needed to identify a specific linking context for weak associations or the precise meaning of an abstract word. The FT subsystem might be associated with semantic control processes that support this function: previous research has suggested that regions sensitive to semantic control demands are situated between FT DMN and multiple-demand network regions on the cortical surface, for example, in the left medial and inferior lateral frontal and posterior temporal cortex (Davey et al., 2016; Wang et al., 2021; Chiou et al., 2023). In line with this, our comparison of intrinsic connectivity for FT and MT DMN showed stronger coupling of the key nodes of the semantic control network—namely left inferior frontal gyrus and posterior middle temporal gyrus—to the FT subsystem (Fig. 6). In this way, semantic control might draw on both DMN and executive control processes to support the ability to tailor conceptual retrieval to suit the circumstances.

There are of course some important limitations of this research. First, we used a restricted range of tasks that focused on semantic cognition; future research is needed to establish if the pattern observed here is replicated in other domains, such as episodic memory and social cognition. Given that we found involvement of all three subsystems in semantic cognition, with variation across them reflecting representational and input processing demands, we might expect parallel findings in any domain in which these cognitive dimensions can be manipulated, in line with our observation that MT supports picture- and scene-based semantic processing as well as episodic memory, while FT supports both reading and autobiographical memory. Another limitation is that different participants were tested on each task; therefore, we cannot explore the extent to which different patterns of recruitment reflect task effects as opposed to individual differences. Studies probing DMN recruitment within the same subjects would be able to establish if there are spatially correlated patterns of activation and deactivation related to perceptual decoupling, visuospatial memory, and abstract cognition across tasks: for example, do patterns in FT linked to verbal versus picture-based semantic retrieval also predict differences between abstract and concrete words? Given that this study relied on reanalysis of published datasets examining semantic cognition, there are many differences between the studies—for example, the data were acquired using different scanners and sequences, the tasks had different structures and lasted for different lengths of time, and data preprocessing was variable, including the smoothing that was applied. For this reason, our analysis examines a series of within-study comparisons of DMN subsystems that are well-controlled for these sources of variability; we do not focus on statistical comparisons between the datasets which would be hard to interpret. Similarly, we focus on connectivity patterns for regions found to commonly activate and deactivate across Studies 1–4, and we are unable to establish whether these effects vary in their location across different experiments. Despite these limitations, our study provides important constraints on theories of DMN functioning.

In conclusion, we show that DMN subsystems play complementary roles in semantic cognition that are related to their distinctive connectivity patterns, captured by their location within a multidimensional space defined by spatially overlapping cortical gradients. None of these DMN variants was task negative; instead, their recruitment varied according to the need to allocate attention to external inputs in service of a task and to represent visuospatial and abstract conceptual information.
